# Geospatial Hotspot Analysis and Endemicity Trends of Missing and Unrecovered Children in India

**DOI:** 10.7759/cureus.39955

**Published:** 2023-06-04

**Authors:** Tejas J, Siva Kumar, Prem S Panda, Ipsita Debata, Thirunaaukarasu D, Balakrishnan Jaya

**Affiliations:** 1 Forensic Medicine, Karpaga Vinayaga Institute of Medical Sciences and Research Center, Chennai, IND; 2 Forensic Medicine, Apollo Institute of Medical Sciences and Research, Chittoor, IND; 3 Community Medicine, Kalinga Institute of Medical Sciences, Bhubaneswar, IND; 4 Community and Family Medicine, Kalinga Institute of Medical Sciences, Bhubaneswar, IND; 5 Community Medicine, Karpaga Vinayaga Institute of Medical Sciences and Research Center, Chennai, IND; 6 Physiology, Karpaga Vinayaga Institute of Medical Sciences and Research Center, Chennai, IND

**Keywords:** self-organizing maps, endemicity, geospatial analysis, unrecovered, kidnapping, children, missing

## Abstract

Background: Children constitute a nation’s true asset. A country's future relies upon the proper development of its children, which necessitates a supportive environment and sufficient opportunities. Children, under the age of 18 years form a considerable percentage of India's population which imparts a huge responsibility for the nation. Everyday we come across news about a child going missing. The National Crime Record Bureau (NCRB) states that 73,138 children were reported missing overall in 2018. The prevalence increased by 8.9% in 2019, a worrisome situation. The cause behind children going missing is multi-dimensional like poverty, unemployment, loss of livelihood, natural calamities, social conflicts, and migration to urban areas. At present, missing children remain a neglected and non-urgent intervention area for everyone. Only the parents whose children are missing can feel the vacuum and sorrow of the situation. The sociologies of India’s missing children merit dimensional and circumstantial examination. The sociological space into which a child goes missing is highly under-researched in India. This study helped in understanding the magnitude of missing cases across India based on existing literature and secondary sources. It also identified the potentially safe and worst areas with regard to missing children. The endemicity helped in identifying the changing trends in each of these areas of interest, which could serve as baseline data for policymakers and law enforcement alike.

Materials and methods: This is a cross-sectional analytical type of study. The data of missing and unrecovered children for the past five years (2021-2017) were obtained from open government data portal (https://data.gov.in) and geospatial hotspot analysis was done using the Getis-Order-G statistics on the same using GeoPandas and PySAL libraries of python. The endemicity of missing cases was studied using hierarchical cluster analysis and self-organizing maps using Python.

Results: For boys, Uttar Pradesh, Rajasthan, and Madhya Pradesh remained consistent hotspots (high risk of missing cases) across all five years of study whereas Karnataka became a hotspot for 2020 and 2021. Among the cold spots (low risk of missing cases), Andaman and Nicobar Islands remained consistent cold spot across all five years of study and has emerged as a safe haven for boys. The missing girls' data reveals that Uttar Pradesh has consistently been the worst state for girls (along with their neighbors) as it possess the risk of girls going missing all through the study period with Chhattisgarh being included from 2019 onwards. Jharkhand, Gujarat, Sikkim, and Andaman and Nicobar Islands have consistently been cold spots across the board and have emerged as areas with low risk for missing girls.

Conclusion: This study helps us in understanding the magnitude of missing cases across India and it also identifies which are potentially safe areas as well as worst areas with regard to missing children. The endemicity also helps us in identifying the changing trends in each of these areas of interest. This will serve as a great resource for policy makers and law enforcement alike.

## Introduction

Children constitute a nation’s true asset. A country's future relies upon the proper development of its children, which necessitates a supportive environment and sufficient opportunities. Children, under the age of 18 years, constitute 39% of the total population in India (Census, 2011). In numbers, it is around 472 million, which is 19% of the world’s children. This gigantic number imparts a huge responsibility for the nation [1]. The phase of childhood is synonymous with innocence, freedom, and joy. They are spared the responsibility and obligations of adult life. It is also the most vulnerable time period. Society as a whole ought to act as the guardians of children and be responsible for their welfare and development. But in reality, not a single day goes by when we do not read about a case in which a child has either been exploited, abused or missing, or killed. Children are susceptible to a range of crimes.

The magnitude of the problem of missing children is as follows: the sad reality is that one child goes missing in India every 8 minutes, according to the National Crime Records Bureau (NCRB). Out of these missing children, as much as 40% of children have never been found, which is very disheartening not only for the parents of those missing children but for the entire nation. The annual "Crime in India - 2019" report from National Crime Record Bureau stated that 73,138 children were reported missing in 2018. The prevalence increased by 8.9% in 2019, a worrisome situation [2].

Who are these missing children? A definition of a missing person on Wikipedia states that “A missing person is a person who has disappeared and whose status as alive or dead cannot be confirmed as their location and fate are not known” [3]. The Association of Chief Police Officers (ACPO) defines a missing person as “Anyone whose whereabouts are unknown whatever the circumstances of disappearance. They will be considered missing until located and their well-being or otherwise established” [4].

The definition of missing children was ambiguous until an advisory was issued by the Ministry of Home Affairs (MHA) on missing children on January 31, 2012. The advisory, the Justice Juvenile (Care and Protection of Children) Act (JJA), 2000, defines a missing child as "a person under 18 years of age whose location is not known to the parents, legal guardians, or any other person who may be legally assigned with the custody of knowing the wellbeing of the child whatever may be the circumstances. The child will be considered missing and in need of care and protection, until located and/or his/her safety/wellbeing is established [5].

Why do these children go missing? Children either run away on their own or are forced to run away due to enthralling circumstances in their families and extended surroundings like children facing hostile environments and are asked to leave home, children who are deserted by irresponsible parents or due to the death of their parents or because of their gender (female child), children who are trafficked and exploited for various purposes like begging, sex trades, child bride, smuggling, etc., children who are lost or injured in mass social gatherings and are unable to find their way back to their parents.

A number of intertwined sociologies create problems like multi-dimensional levels of unemployment, poverty, communal conflicts, loss of livelihood, natural calamities, and migration to urban areas [6-9]. Thus, each group of children typifies different social problems. Because these groups of missing children are so heterogeneous, there is no particular data or established definitions to describe them. Another question to ponder is why reports of missing children are not being treated as cognizable offense. When a child goes missing, there is no structured way of registering an FIR across the country [10]. Complaints of missing children are treated like any other non-cognizable offense and only an entry is made in the General Station Diary (GD), which is then followed by an inquiry.

The aim of this study is discussed further. Currently, missing children remain an under-researched intervention area for everyone. Only the parents whose children are missing can feel the vacuum and sorrow of the situation. The sociologies of India’s missing children merit dimensional and circumstantial examination. The sociological space into which a child goes missing is highly under-researched in India. This study will help in understanding the magnitude of missing cases across India based on existing literature and secondary sources. It will also identify the potentially safe and worst areas with regard to missing children. The endemicity will help in identifying the changing trends in each of these areas of interest, which will serve as baseline data for policymakers and law enforcement alike. 

## Materials and methods

This was a cross-sectional analytical type of study of missing children in India. The data consisted of state-wise missing boys and girls across India for the past five years, ranging from 2017 to 2021. This secondary data, published by the National Crime Records Bureau of India, was obtained from the open-source government data publication portal. The data was cleaned, sorted sate-wise, filtered year-wise, and segmented to separate boys from girls using MS Excel. The Excel files thus generated were imported into Jupyter Notebook to run the geospatial analysis using Python v3.9.11. The Python libraries used for the geospatial analysis included NumPy v1.23.4, Pandas v1.5.1, GeoPandas v0.12, PySAL 2.4.3, and scikit-learn v1.1.3. The exploratory geospatial analysis was performed with GeoPandas. The spatial auto-correlation and geospatial hotspot analysis using the Moran Cluster technique was performed on the same data using PySAL library to get hot and cold spots for the missing children across India state-wise [11]. To study the endemicity pattern of missing children, data were analyzed using hierarchical cluster analysis to compute cluster sizes and generate Self Organizing Maps using that cluster size with the help of self-organizing map (SOM) module in scikit-learn library. Results have been presented as tables and its findings are discussed. The maps for hot/cold spot analysis have depicted hot spots in red and cold spots in light blue.

## Results

The findings of our study with respect to missing boys during the years 2017-2021 have been depicted in Figures 1-5 and Table 1. The findings of our study with respect to missing girls during the years 2017-2021 have been depicted in Figures 6-10 and Table 2.

**Figure 1:**
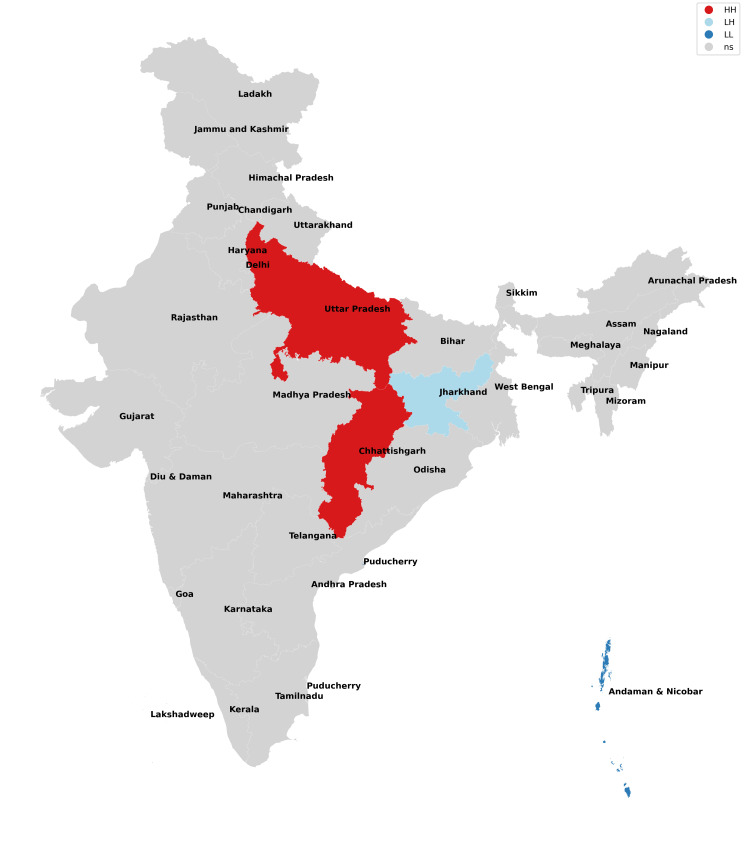
Moran cluster map of missing boys in 2017.

**Figure 2:**
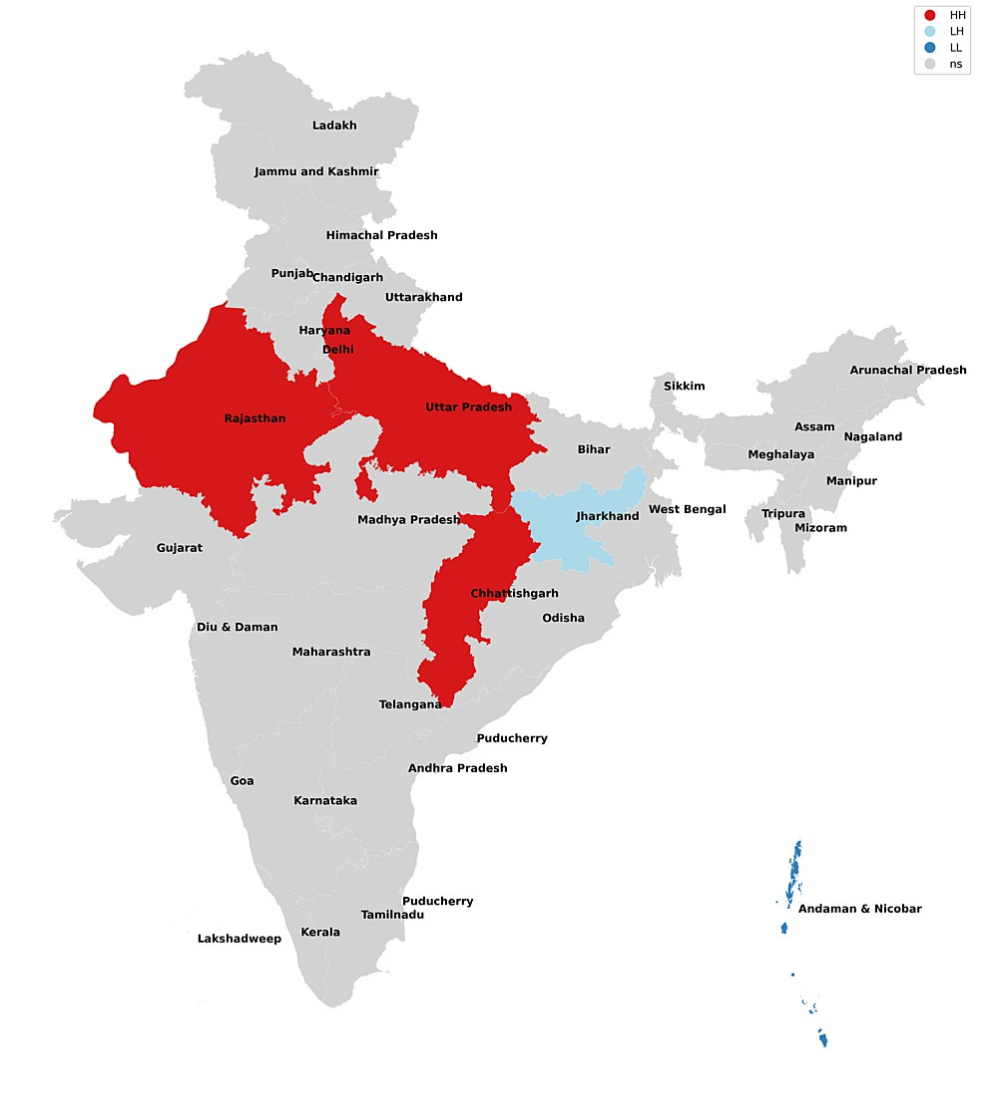
Moran cluster map of missing boys in 2018.

**Figure 3:**
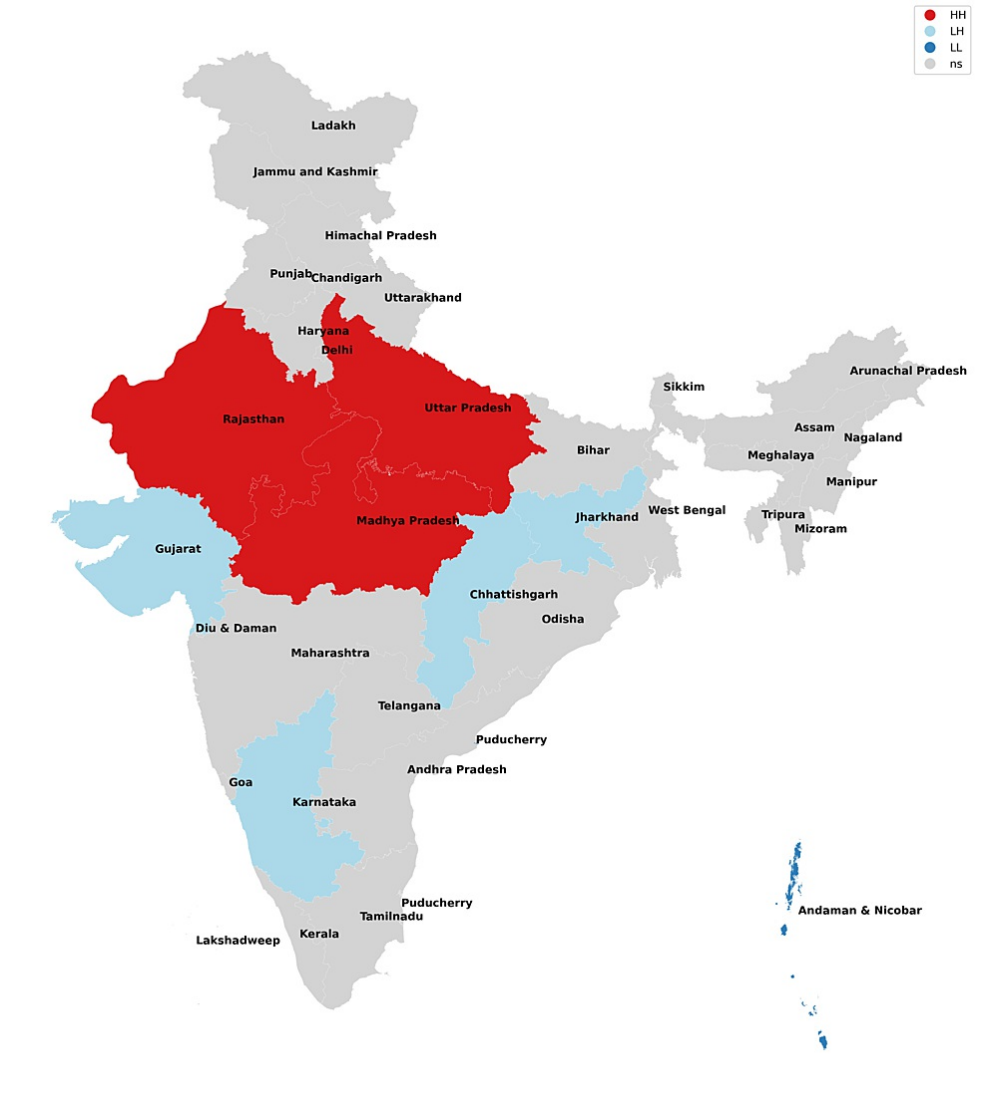
Moran cluster map of missing boys in 2019.

**Figure 4:**
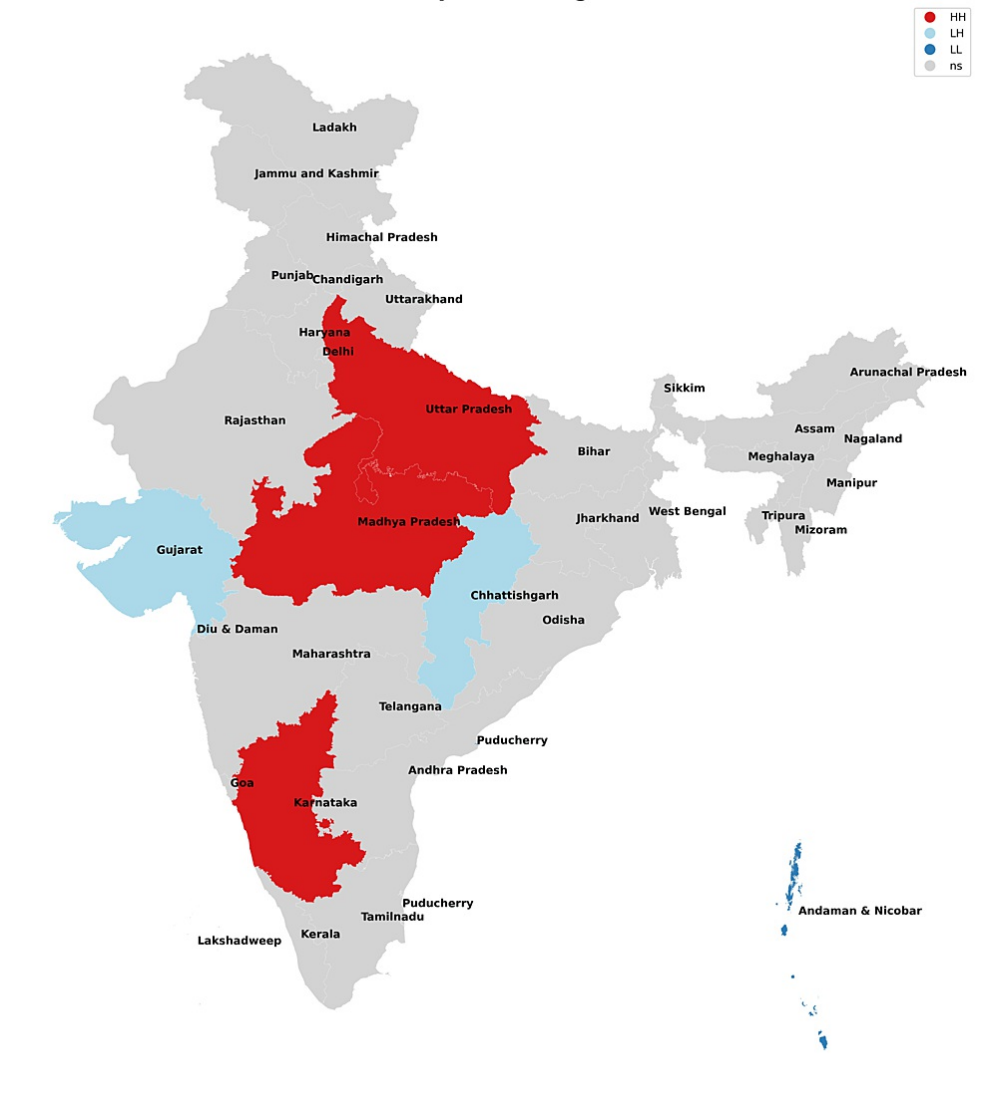
Moran cluster map of missing boys in 2020.

**Figure 5:**
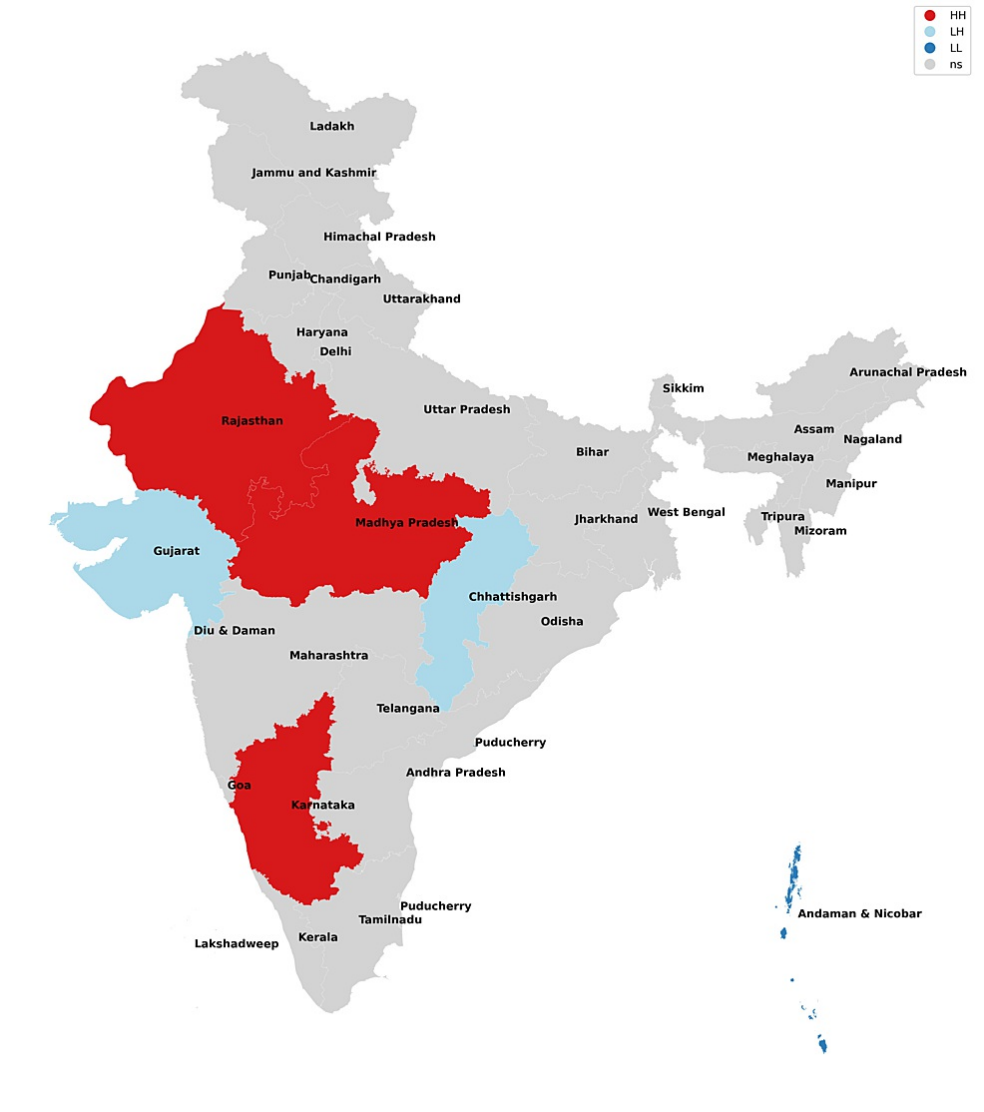
Moran cluster map of missing boys in 2021.

**Table 1:** Hot and cold spot analysis results for missing boys from 2017 to 2021.

Year	2017	2018	2019	2020	2021
Total missing boys	20,555	19,784	21,074	13,566	17,977
Hot spots (n, %)	Uttar Pradesh (1,433, 6.97%), Chhattisgarh (629, 3.06%)	Rajasthan (666, 3.37%), Uttar Pradesh (1,341, 6.78%), Chhattisgarh (693, 3.5%)	Uttar Pradesh (1,283, 6.09%), Madhya Pradesh (2,450, 11.63%)	Madhya Pradesh (1,521, 11.2%), Karnataka (471, 3.47%)	Rajasthan (803, 4.47%), Madhya Pradesh (2,200, 12.24%), Karnataka (562, 3.13%)
Cold spots (n, %)	Jharkhand (209, 0.01%), Andaman and Nicobar Islands (13, 0.06%)	Andaman and Nicobar Islands (10, 0.05%)	Chhattisgarh (537, 2.54%), Andaman and Nicobar Islands (10, 0.05%,)	Gujarat (289, 2.1%), Chhattisgarh (352, 2.6%), Andaman and Nicobar Islands (7, 0.05%)	Gujarat (344, 1.91%), Chhattisgarh (413, 2.23%), Andaman and Nicobar Islands (5, 0.03%)

**Figure 6:**
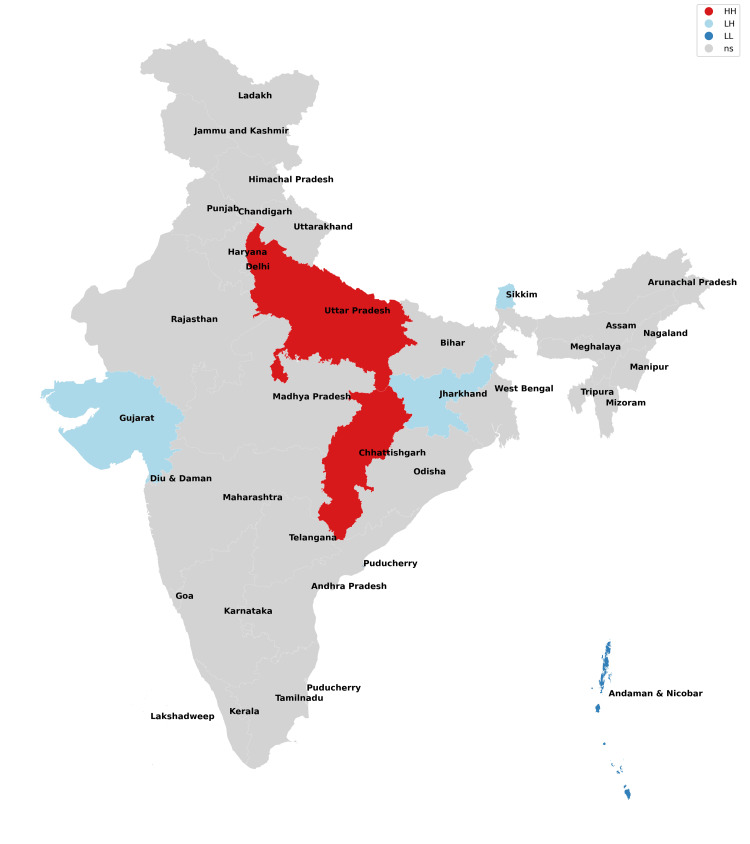
Moran cluster map of missing girls in 2017.

**Figure 7:**
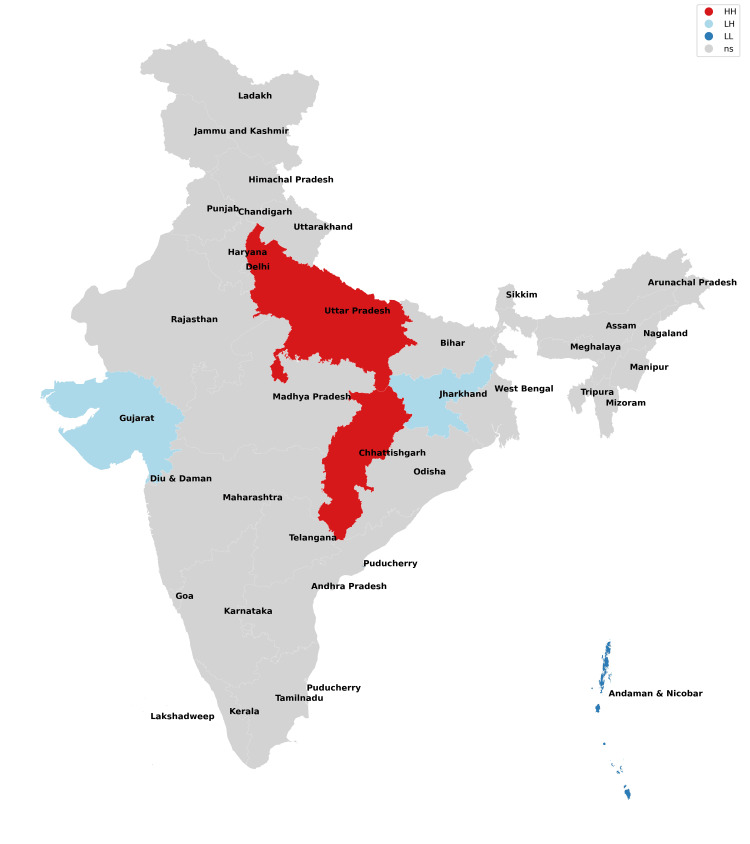
Moran cluster map of missing girls in 2018.

**Figure 8:**
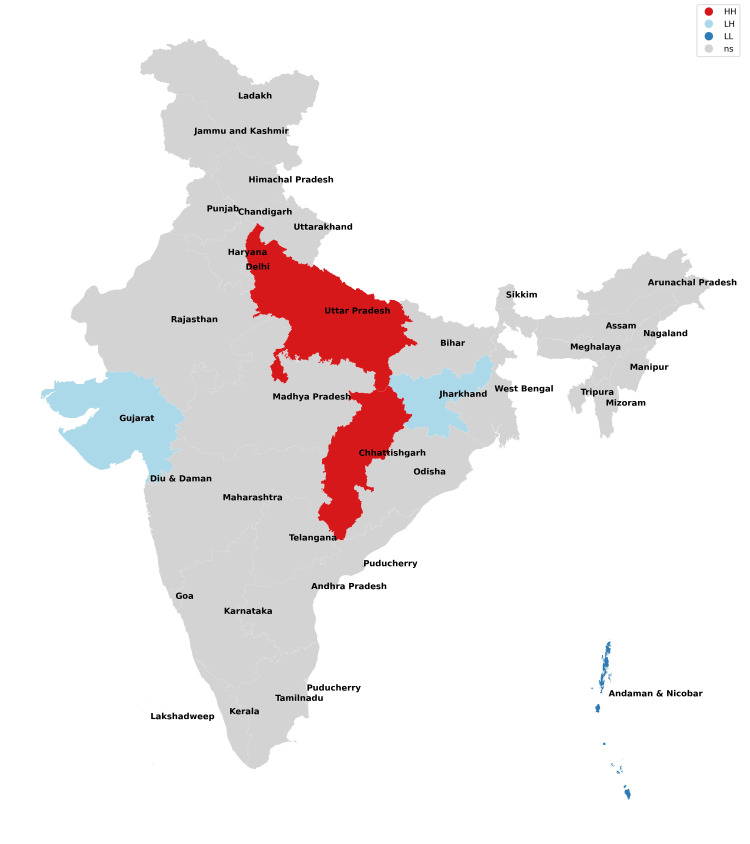
Moran cluster map of missing girls in 2019.

**Figure 9:**
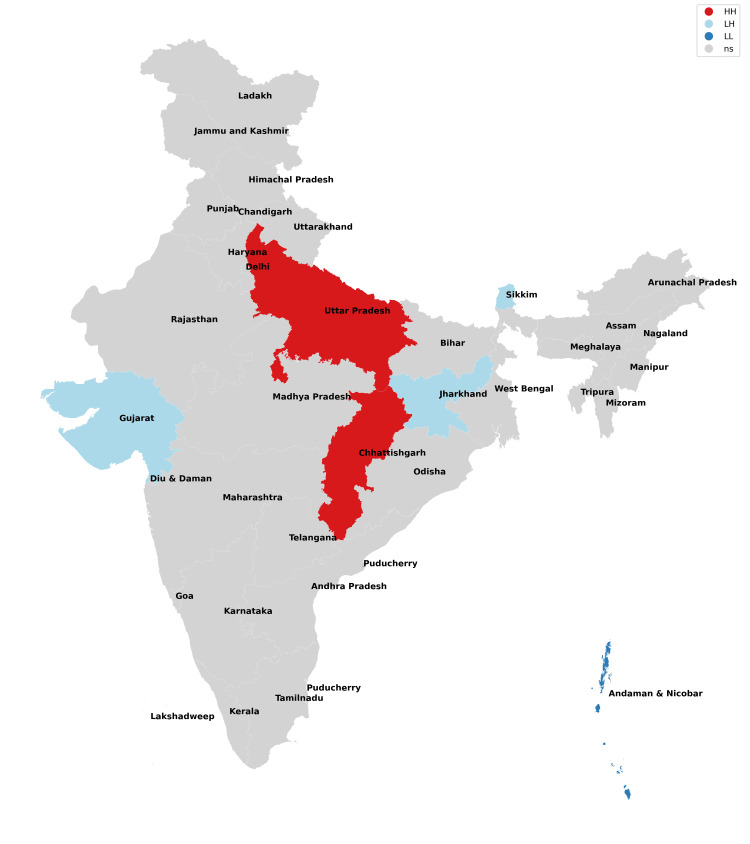
Moran cluster map of missing girls in 2020.

**Figure 10:**
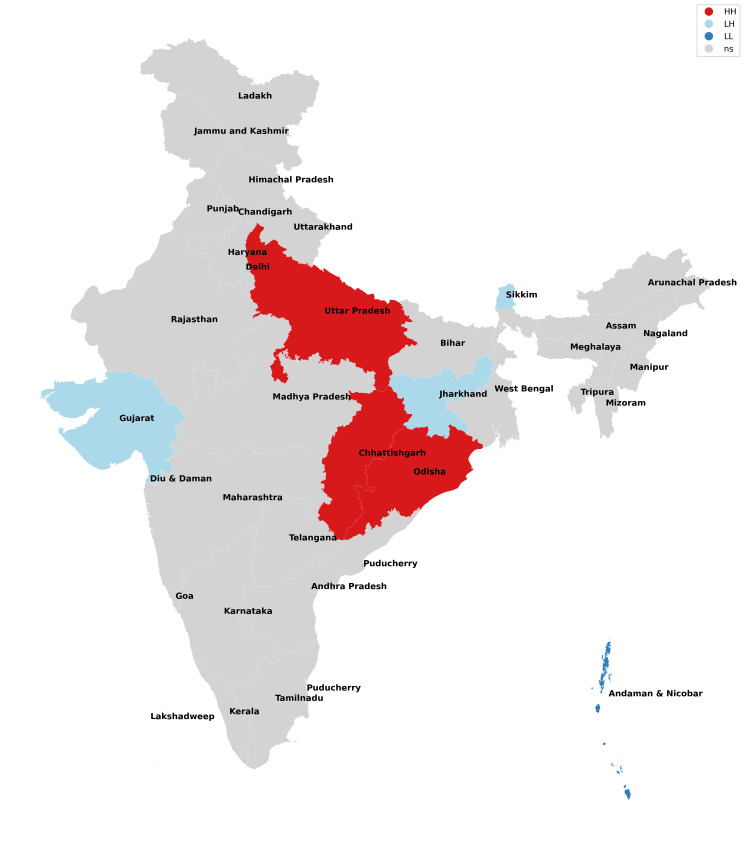
Moran cluster map of missing girls in 2021.

**Table 2:** Hot and cold spot analysis results for missing girls from 2017 to 2021.

Year	2017	2018	2019	2020	2021
Total missing boys	42,711	47,225	52,085	45,716	59,569
Hot spots (N, %)	Uttar Pradesh (1,526, 3.5%)	Uttar Pradesh (1,965, 4.16%)	Uttar Pradesh (1,901, 3.64%), Chhattisgarh (2,606, 5%)	Uttar Pradesh (1,469, 3.21%), Chhattisgarh (2,107, 4.74%)	Uttar Pradesh (2,168, 3.64%), Chhattisgarh (2,865, 4.81%)
Cold spots (N, %)	Jharkhand (211, 0.4%), Gujarat (1,025), Sikkim (13, 0.03%), Andaman and Nicobar Islands (25, 0.05%)	Jharkhand (203, 0.43%), Sikkim (24, 0.05%), Andaman and Nicobar Islands (38, 0.08%)	Jharkhand (170, 0.32%), Gujarat (1,044, 2%), Andaman and Nicobar Islands (33, 0.06%)	Jharkhand (202, 0.44%), Gujarat (915, 2%), Sikkim (11, 0.02%), Andaman and Nicobar Islands (14, 0.03%)	Jharkhand (245, 0.41%), Gujarat (1,049, 0.41%), Sikkim (6, 0.01%), Andaman and Nicobar Islands (20, 0.03%)

**Figure 11:**
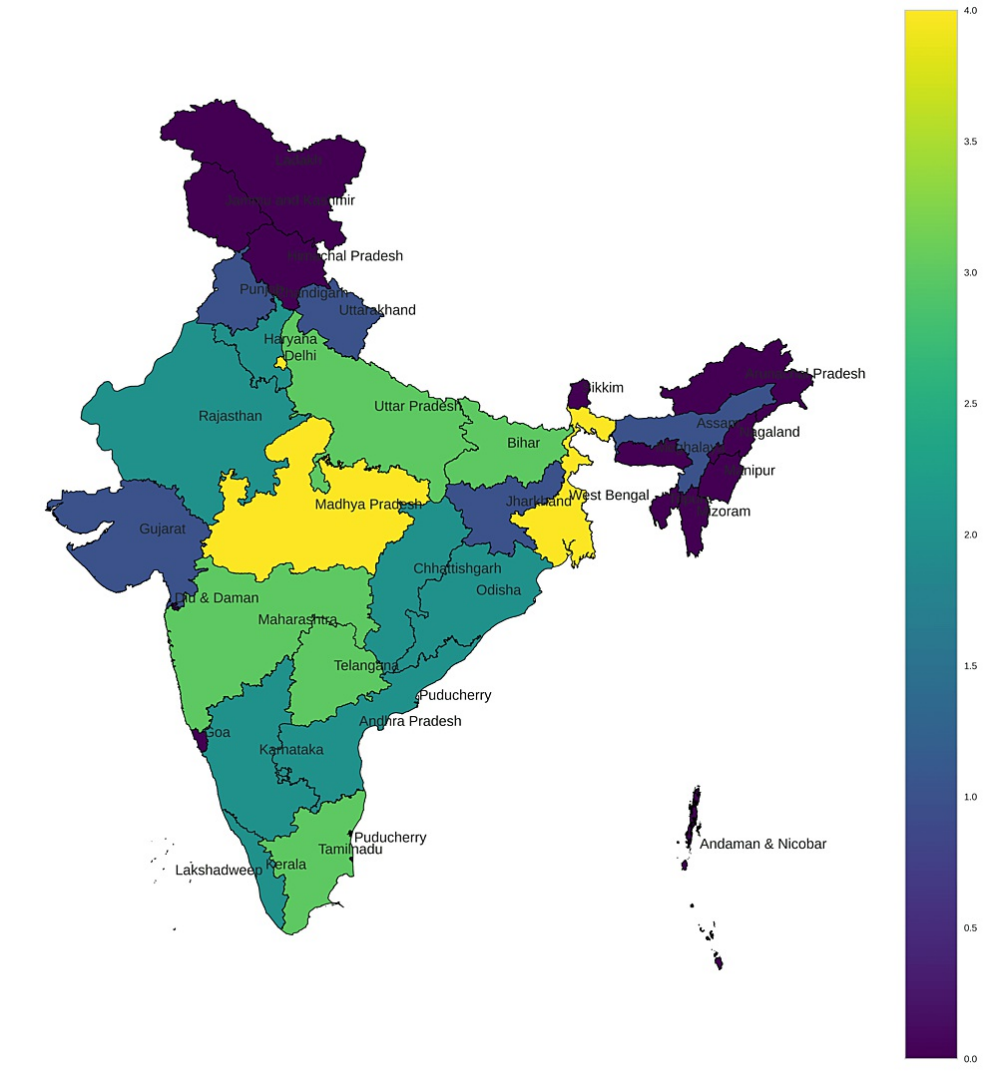
Self-organizing map of missing boys from 2017 to 2021.

**Figure 12:**
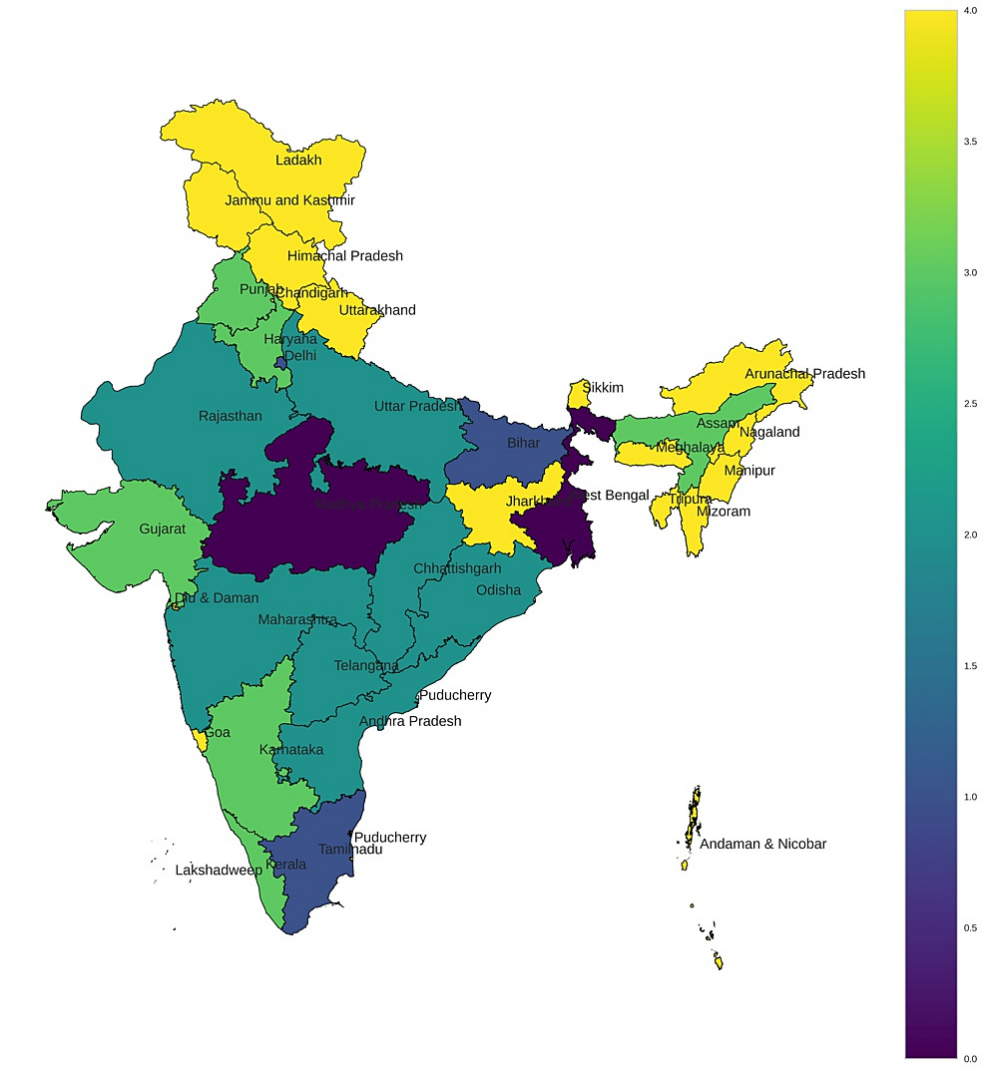
Self-organizing map of missing girls from 2017 to 2021.

Hierarchical cluster analysis was performed independently on missing boys’ and missing girls’ data and an optimum cluster size of 5 was chosen using the Elbow method for both sexes. Data were further subjected to self-organizing map analysis with a cluster size of 5 and results were displayed in map E1 and map E2 for boys and girls, respectively (Figures 11, 12 and Tables 3, 4).

**Table 3:** Endemicity clustering using self-organizing maps for missing boys.

Clusters	States
0 (high prevalence)	Madhya Pradesh, West Bengal, Delhi
1	Bihar, Maharashtra, Tamil Nadu, Telangana, Uttar Pradesh
2	Andhra Pradesh, Chhattisgarh, Haryana, Karnataka, Kerala, Odisha, Rajasthan
3	Assam, Gujarat, Jharkhand, Punjab, Uttarakhand
4 (low prevalence)	Arunachal Pradesh, Goa, Himachal Pradesh, Manipur, Meghalaya, Mizoram, Nagaland, Sikkim, Tripura, Andaman and Nicobar, Chandigarh, Diu and Daman, Jammu and Kashmir, Ladakh, Lakshadweep, Puducherry

**Table 4:** Endemicity clustering using self-organizing maps for missing girls.

Clusters	States
0 (high prevalence)	Madhya Pradesh, West Bengal
1	Bihar, Tamil Nadu, Delhi
2	Andhra Pradesh, Chhattisgarh, Maharashtra, Odisha, Rajasthan, Telangana, Uttar Pradesh
3	Assam, Gujarat, Haryana, Karnataka, Kerala, Punjab
4 (low prevalence)	Arunachal Pradesh, Goa, Himachal Pradesh, Jharkhand, Manipur, Meghalaya, Mizoram, Nagaland, Sikkim, Tripura, Uttarakhand, Andaman and Nicobar, Chandigarh, Diu and Daman, Jammu and Kashmir, Ladakh, Lakshadweep, Puducherry

## Discussion

In this analysis, the missing boys' data reveal that Uttar Pradesh, Rajasthan, and Madhya Pradesh remained consistent hotspots (high risk of missing cases) across all five years of study, whereas Karnataka became a hotspot for 2020 and 2021 which implies that these states and their neighbors have been most unsafe for boys (Figures 1-5, Table 1). Chhattisgarh was a hotspot for 2017 and 2018 but remained a cold spot for the rest of the years of study. Among the cold spots (low risk of missing cases), Andaman and Nicobar Islands remained a consistent cold spot across all five years of study and have emerged as a safe haven for boys. Jharkhand was a cold spot only for 2017. Gujarat emerged as a cold spot from 2020 onwards. Such observations have not been researched previously as the majority of studies focus on missing girls and women in general.

The missing girls' data reveals that Uttar Pradesh has consistently been the worst state for girls (along with their neighbors) as it possesses the risk of girls going missing all through the study period with Chhattisgarh being included from 2019 onwards (Figures 6-10, Table 2). These findings are consistent with the study of Sarkar who reported a rise in women and children being trafficked from above places for commercial exploitation and sex trafficking and the following falsities have been widely used for trafficking in women and children in India: (a) offering jobs as domestic help, (b) promising jobs in acting, (c) promising position in factories, (d) lending money, (e) luring them with jaunt journey, (f) fake promises for marriage, (g) befriending them by giving dainties, (h) offering pilgrimage trips, (i) other kinds of false promises, and (j) intimidation [12].

Bride trafficking is particularly evident in Haryana because of its dismal sex ratio [13]. Jharkhand, Gujarat, Sikkim, and Andaman and Nicobar Islands have consistently been cold spots across the board and have emerged as areas with low risk for missing girls. Ghosh also observed similar findings but reported the need for a comprehensive program for managing trafficking of women and children, protection, rehabilitation, and reintegration of rescued victims into the study and also raised doubts regarding the authenticity of data of missing victims as the states which shared international borders did not have a robust reporting mechanism [14].

High endemicity for missing boys was seen in Delhi. Madhya Pradesh and West Bengal remained highly endemic for both sexes (Figures 11, 12, Tables 3, 4). The northeastern states of India and union territories showed low endemicity for missing children in general which could be due to their relatively modest population and lack of neighbors in case of Islands like the Andaman and Nicobar.

On June 2, 2015, the Ministry of Women and Child Development launched its second website, khoyapaya.gov.in (lost and found), where a person can fully participate in uploading information about a missing child, report detection, or browse the database to retrieve information about a lost child [15]. Even though Khoya-Paya makes timely reporting of missing children more convenient and is definitely a promising development; awareness about this portal needs to be created via mass media and among the lower classes of society whose children remain at high risk. But the utility of such services has not been widely publicized or put to good use.

## Conclusions

In our study, Uttar Pradesh, Rajasthan, and Madhya Pradesh were identified as the unsafe states where missing cases of boys were more prevalent across all five years of study. Andaman and Nicobar Islands and Gujarat emerged as safe haven for boys. Uttar Pradesh again with Chhattisgarh was most unsafe for girls while Andaman and Nicobar Islands, Jharkhand, Sikkim, and Gujarat were states with the lowest prevalence of missing girls. High endemicity for missing boys was seen in Delhi. Madhya Pradesh and West Bengal remained highly endemic for both sexes. The northeastern states of India and union territories showed low endemicity for missing children in general. This study helps us in understanding the magnitude of missing cases across India. It also identifies the potentially safe areas as well as the worst areas with regard to missing children. The endemicity also helps us in identifying the changing trends in each of these areas of interest. This will serve as a great resource for policymakers and law enforcement alike.
